# Research on Outdoor Space Design Strategy of “SOS Children's Village” Based on the Psychology of Troubled Children

**DOI:** 10.3389/fpubh.2022.870288

**Published:** 2022-05-02

**Authors:** Fei Fu, Xiao Liu, Sixiang Zhou

**Affiliations:** School of Architecture, Southwest Jiaotong University, Chengdu, China

**Keywords:** children in distress, outdoor space, child psychology, SD method, design strategy

## Abstract

Despite significant interaction between children's psychology and space in welfare institutions, only a handful of empirical studies have focused on it to date. Through the investigation, analysis, and spatial classification of the psychological issues of troubled children in the SOS children's village community in Chengdu, we observed the records for 1 year and used the PHCSS-SD method innovatively to systematically and quantitatively analyze the psychology of troubled children and their perception of public space in the park. Based on the needs of hearing, vision, touch, interaction, and safety of children in distress, we selected nine evaluation factors, including daylighting, interesting pattern, participation, and touchability. Each factor was categorized into five evaluation scales for psychological measurement and analysis to provide a basis for the development of mental health and the optimization of the living environment of children in distress. Based on the SD broken line of public space, we analyzed the advantages and disadvantages of space and affirmed the crucial contributions made by the Chinese government to children's welfare. Overall, this study discusses the strategy of building outdoor public spaces in the SOS children's village community.

## Introduction

**Figure 1:**
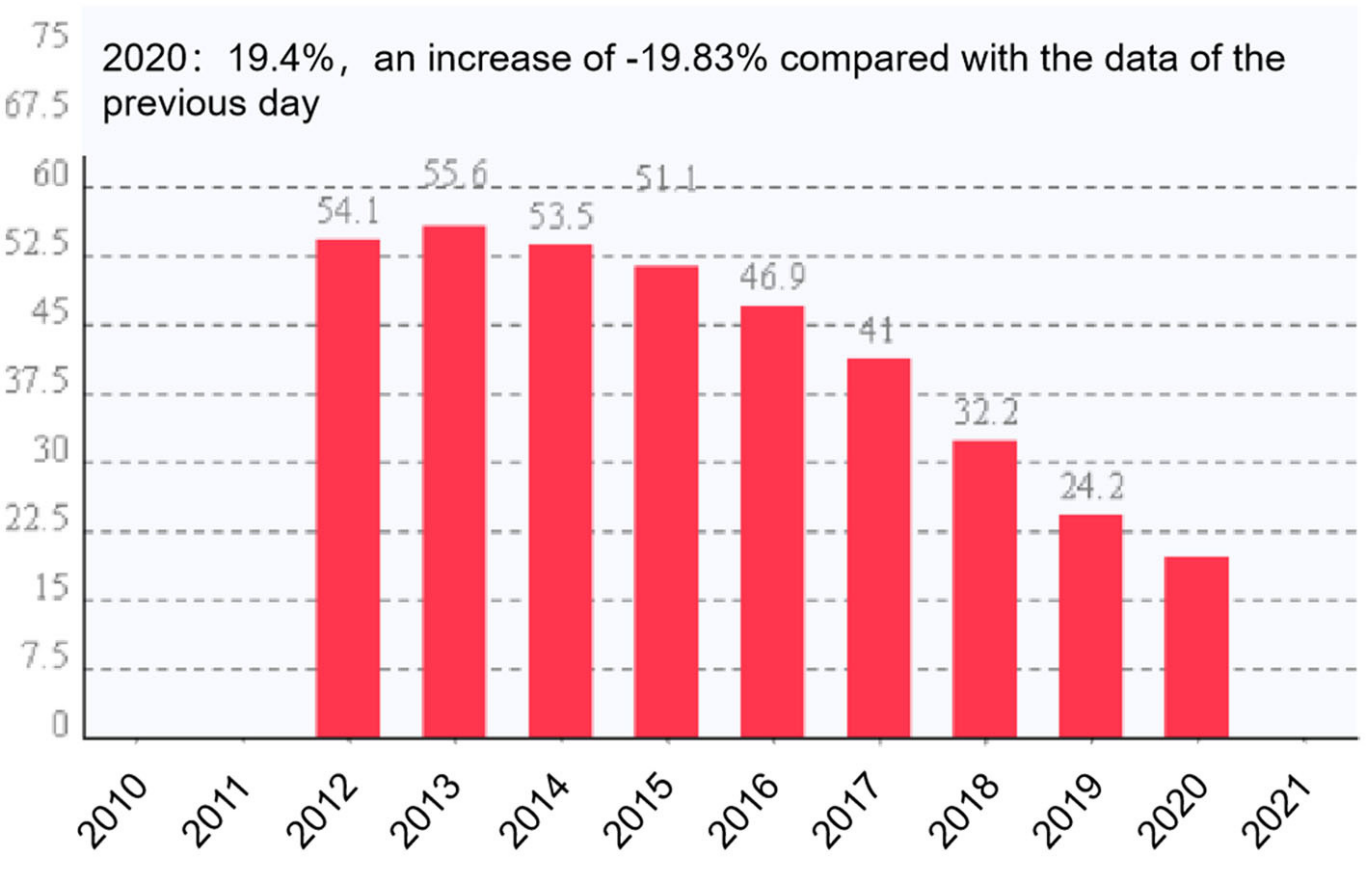
Statistics of children in distress in China in 2021.

Since the reform and opening-up, China has experienced a remarkable transformation (1–3). As a vulnerable group, children are in a transitional society. Fronting the complex situation, their growth exhibits a diversified trend, and they are easy to fall into the dilemma of mobility, inequality, and instability (4). At the end of 2020, the law of the People's Republic of China on the protection of minors and the prevention of juvenile delinquency was revised and promulgated successively, and the professional role of social welfare work was established by legislation. Previously, the proposal of the CPC Central Committee on formulating the 14th five-year plan for national socioeconomic development and the long-term goals for 2005 proposed the requirements to protect the legitimate rights and interests of women and children and upgrade the social welfare system for orphans. In recent years, the government has continuously increased public expenditure on children in distress, including cash expenditure, government purchases, and support to social institutions. With the government's attention, the number of children in distress in China has also decreased yearly to 210,000 this year (Figure 1).

However, a large number of children remain in distress in China. According to the 2021 China Civil Affairs Statistical Yearbook—China social service statistics, there were 240,000 orphans in 2020, with 169,000 social scattered orphans and 2.24 million children in poverty. Moreover, countless children with severe disabilities, rare diseases, and abuse are experiencing inconceivable problems such as survival, safety, schooling, medical treatment, and many more. Children negotiate, struggle, and adapt to the external and internal world in their own way (5). Thus, paying attention to the construction of community communication space plays a vital role in the physical and mental health development and self-worth realization of children in distress. China is gradually attaching more importance to child welfare protection, and social development is also presenting higher requirements for all children in distress (6). With the snowballing number of children in distress, the demand for quality of life is also increasing (7). Nevertheless, the imperfect community service system, low participation of residents, intensification of commercialization tendency (8), and the alternative neglect of traditional ideas to vulnerable groups lead to deficient children's services. Children's growth space is constantly compressed, and there is a spatial trend of “weak standard,” “skewness,” and “separation” (9). Children's lack of natural perception and the decline of their independent mobility exert negative effects that are not conducive to their physical and mental growth (10). Meanwhile, common problems exist in the overall space environment, targeted design, and humanized treatment (11, 12).

Owing to the rapid urbanization, people are facing the lack of significant outdoor activity places, compressing users' communication space and time. As a vulnerable group, children are more prone to forming an outlier character of loneliness, introversion, and poor communication. “Children in trouble” is even worse, as they lack the protection of society and family, lose their dependence, and are in trouble after family collapse events. In addition, they have serious physical and psychological defects. Unlike regular children, those in distress reflect great differences in psychophysiology, not only at the material level but also in all aspects of the spiritual world and growth process. The psychological issues of troubled children, as a disease of psychological disability, render them more vulnerable to life ability and have higher instability (13).

Children in distress often play in outdoor spaces. As a learning way of exploration and experience, games are not only a game space but also a learning place for them. In recent years, the high mobility of the travel environment has directly declined children's independence and even various adverse symptoms such as autism and obesity (14). A data survey revealed that the sedentary problem of Chinese children in recent years led to their serious lack of activity, causing great harm to their health. Besides, there are multiple obstacles to the psychological problems and social interaction of children in distress. To stimulate the sense of belonging and self-identity of children in distress, the outdoor space design of children's community is of utmost significance in the special period of their growth, and it is also the unshirkable responsibility of the whole society (15). The optimization of family structure in the welfare community of the SOS children's village can boost the behavior activities of children in distress (16). The outdoor activity space of the children's village is the place where children in distress have the most frequent communication behavior, except at school. As the fundamental unit constituting the spatial support system of children's friendly city, it is the “first environment” for them to exercise their rights. Hence, the impact of space construction on children's communication behavior cannot be overlooked (17).

Based on the summary and induction of scholars' research on welfare community for children in distress in recent years, this paper puts forward the elements of research on public activity space of welfare community, analyzes the living situation of children in distress, and puts forward the renewal strategy of public space of welfare community based on the concept of healing nature. It has profound social significance to awaken the social and humanistic care for children in distress, enhance the sense of belonging and self-identity of children in distress, and effectively solve the psychological problems of children in distress. In addition, the comprehensive use of social assistance, social welfare, security and other policies and measures, classified implementation of policies and precise assistance, to create a demonstration environment for the healthy growth of children in distress for the promotion and learning of SOS Children's villages across the country, and also a response to the call of national policies.

## Children's Psychological Theories and Behavioral Characteristics

### Overview of Environmental Space From the Perspective of Children

In 1924, the 5th United Nations General Assembly adopted the Geneva Declaration on the rights of the child, which proposed: “children must be provided with all kinds of material and spiritual needs.” In 1933, the urban planning outline in the Athens Charter stipulated that attention should be paid to the composition of children's living and activity space, and proposed: “in new communities, open space should be reserved for the construction of parks, playgrounds and children's activity sites.” In 1959, the 14th United Nations General Assembly adopted the declaration on the rights of the child, highlighting: “relevant laws and regulations should be formulated to protect children's rights under the principle of maximizing children's interests” and “children have the right to education, play and entertainment.” Joe L. Frost, in his book *Play and Child Development*, proposed that paying attention to children's natural amusement tendency is a key principle of venue design and proposed the significance of children's autonomous activity venue. The “Outdoor Space for Child Care” of Human Places: Guidelines for Urban Open Space Design compiled by Clare Cooper Marcus and Carolyn Francis mentions that the design of outdoor activity space can support active game activities to promote children's physical development. Jane Jacobs highlights in the *Death and Life of American Big Cities* that children need opportunities to communicate with their living environment. Absolute isolated protection places are not good for children. Society should pay more attention to the interaction between children and others in the process of growth, which is positive and beneficial to their growth. To promote the construction of a child-friendly city, the Portland government of the United States proposed the concept of “complete community,” emphasizing addressing the demand of families with children for community housing and public service facilities. Subsequently, the regulatory guidelines for high-density family settlements with children were formulated, which outlined requirements for public spaces suitable for children in settlements (18).

In China, in 1920, Chen Heqin, a child educator, took his son Chen Yiming as the research object and used the method of diary description and recording to publish China's first monograph on children's psychology “Research on children's psychology” for 3 years. Chen Heqin argued that appropriate play behavior could promote children's physical and mental health development. Huang Yi explored children's language development and character evaluation and authored *The Psychology of Children's Painting*. *The Psychology of Children* published in 1966 examines the development and mechanism of children's psychological process and also refers to the correlation between children and the environment. Regarding the urban social environment, Prof. Yang Guiqing of Tongji University published the book “*Urban Social Psychology*” in 2000 and presented relevant discussions on the relationship between children's growth process and the urban space environment. Among them, behaviorism theory and sociological theory emphasize the role and influence of the acquired environment. Supporting the enhancement of the acquired environment will play a more positive role in shaping children's behavior and psychology, as well as theoretically support that the design of children's activity places should be based on the scientificity and necessity of children's psychological development. In 2009, Li Yuanyuan published a study on the coupling between children's outdoor game venue design and children's behavior and psychology. Aiming at vulnerable children, this paper reported that the research and design of outdoor activity venues should focus on safety. As children's body shape and physical fitness are lower than adults, they should fulfill the requirements in terms of activity space scale. The above-related contents mostly focus on the impact of residential landscape material environment on children's games. This study reviews only 17 academic papers in this direction, as only few papers have focused on the interaction between children's psychology and space in welfare institutions.

### Division of Children Groups

#### Age Division

The United Nations Convention on the rights of the child divides the age of children into 0–18 years. In psychology, according to the research focus and operability, children's ages are categorized as follows: fetal period: conception to birth; early childhood: 0–6 years; middle childhood: 6–12 years; adolescence: 12–18 years (19). Considering the specific situation of children in distress, at present, there are 103 children in the SOS children's village, including 13 children in distress, 2 in extreme poverty, and 88 general orphans; their age distribution is 2–16 years, including 74 children aged 6–12 years, accounting for the majority. During this period, children are sensitive to the outside world and full of learning enthusiasm. Thus, this study only focused on children in the early and middle stages (0–12 years) to examine the influence of external factors on this group in outdoor space activities.

#### Gender Division

The development of children's gender consciousness can be categorized into three stages—fuzzy stage, sensitive stage, and differentiation stage. Different stages of gender awareness exhibit behavioral differences. It is challenging for children in the fuzzy period before the age of 2 years to distinguish their own gender from others. Over time, children's gender cognition becomes increasingly concrete and they can gradually distinguish gender. Girls tend to have stable peer relationships. Owing to their relatively weak identity, they are often easy to obtain help from others and establish a continuous sense of security in life. Thus, the sense of relationship stability is higher than that of boys (20, 21); that is, girls have a higher sense of control over life than boys. When children are in the differentiation stage, great differences exist in the behavior of boys and girls. Boys are more outgoing and tend to stimulate, a broad, and challenging activity environment, while girls prefer introverted and low-energy activities (22).

#### Scale Change

**Figure 2:**
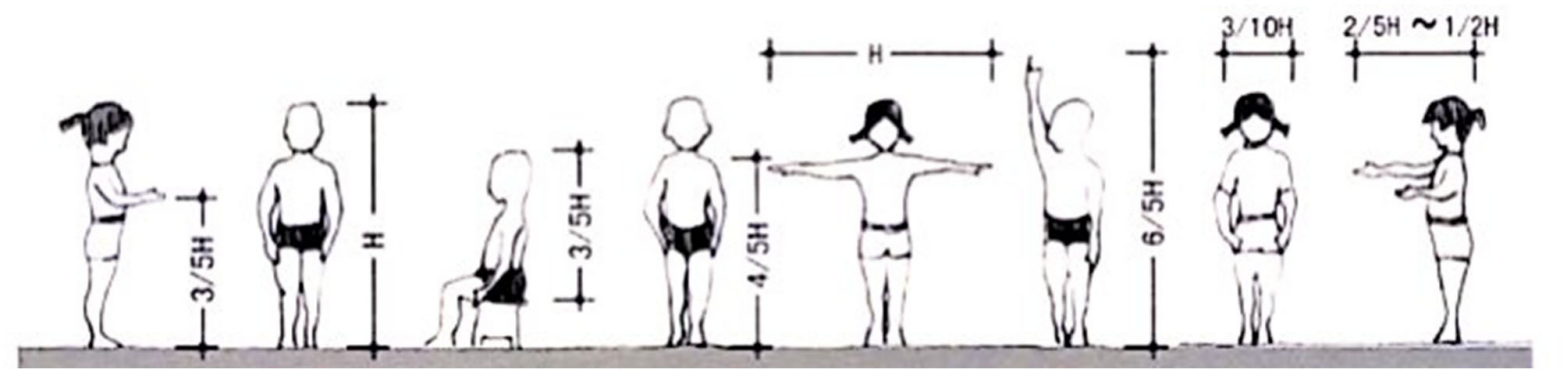
Child scale.

Children's physical characteristics are the basis of children's outdoor activity space planning and design, and children's growth and development scale are a process (Table 1, Figure 2).

**Table 1:** Height and size table of difficult children of different ages in the “SOS children's village.”

**Age**	**Male**		**Female**	
	**Weight (kg)**	**Height (cm)**	**Weight (kg)**	**Height (cm)**
**Average**
3	13.95	95.1	13.44	94.2
4	15.61	102.1	15.21	101.2
5	17.39	108.6	16.79	107.6
6	18.30	111.6	17.92	110.8
8	19.95	116.9	19.23	115.5
10	21.81	132.2	20.95	130.3
12	23.25	150.2	22.46	146.2

With the continuous change of children's height and weight, the range of activities is also expanding. German urban planning highlights that infant activity venues should be set around parents, need adult auxiliary activities, and like imitation and experience, the activity area of school-age children should be 300–400 m; and children should move within the sight of adults and have strong individual expression. The average height of senior children is ~1.5 m. They can move independently and like to gather information. Thus, the length and width of children's outdoor activity site should be more than 1 m, and the use area should be >2.5 m^2^. According to the body growth size data of children in distress, the average growth of children in distress has been found to be the slowest on the scale of 3–5 years.

## Research Methods

### Questionnaire Survey Method

#### PHCSS Children's Self-Awareness Scale

The PHCSS children's self-awareness scale was formulated by Piers Harris in 1969. Its main function is children's self-consciousness evaluation and pathological tracking. Through this table, we can comprehend the views of children in distress on appearance, emotion, study, behavior, happiness, and satisfaction, which is conducive to discovering the deviation of children's psychological self-consciousness and correcting it in time. The scale has 80 questions and is divided into five scales. The total score of children's self-consciousness was assessed according to the national norm and urban norm of Chinese children's self-consciousness scale (total score < 46, low self-awareness; total score > 58, high self-awareness; score = 46–58, normal self-awareness).

#### Eysenck Personality Questionnaire

We adopted the Chinese version revised by Gong Yaoxian of Hunan Medical University. The Eysenck Personality Questionnaire (EPQ) was compiled by Prof. Eysenck, a British psychologist, who collected a large number of non-cognitive characteristics, summarized three orthogonal dimensions through factor analysis and proposed three basic factors determining personality: introversion and extroversion (E), neuroticism (also known as emotional) (n), and psychoticism (P). Different tendencies and degrees of expression in these three aspects constitute different personality characteristics. EPQ is one of the most broadly used questionnaires in the fields of medicine, justice, education, and psychological counseling. The questionnaire has 88 items, including four subscales: extroversion (E), psychoticism (P), neuroticism or emotion (n), and concealment (L).

The general interpretation of the EPQ scale score results is as follows (in fact, it should be calculated and determined according to the standard deviation):

① E scale score: a score >15 indicates an extroverted personality, which could be sociable, eager for stimulation and adventure, and easy to be impulsive. If the score is <8, it means that you are introverted, such as quiet, introspective, do not like stimulation, like an orderly lifestyle, and have relatively stable mood.
② N scale score: a score >14 indicates anxiety, depression, strong emotional response, and even irrational behavior; however, <9 indicates emotional stability.
③ P scale score: if the score is >8, it may be lonely, indifferent to others, difficult to adapt to the external environment, inhumane, unfriendly to others, like to make trouble, like to do strange things, and regardless of danger.
④ L scale score: if the score is >18, it shows that the subjects tend to cover up, and the test results could be distorted.

**Figure 3:**
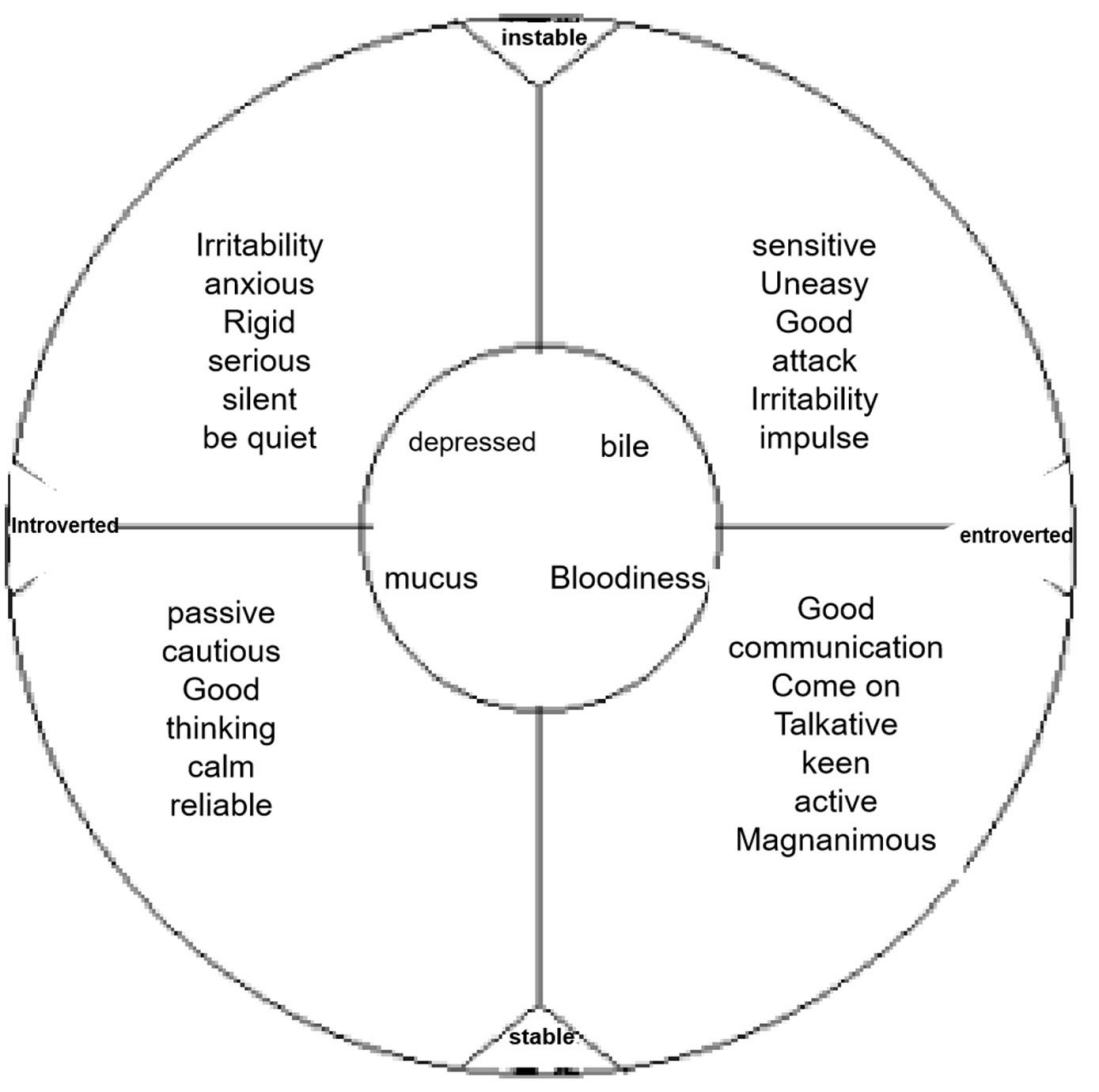
Eysenck personality questionnaire subscale. Source: medical psychology.

According to the total score (rough score) obtained by subjects on each scale and the standard score *t* (*t* = 50 + 10 × (*x* – m)/SD) converted according to the norm, we can analyze the results of the personality characteristics of children in distress as subjects. The average score of the tested population and the total score of the tested population are expressed as the standard deviation (the score of *X* and *m* are expressed as the standard deviation; Figure 3).

### SD Methods

The SD method selects as many adjective pairs related to describing the research object as possible. This method has been extensively used in the assessment of urban design, landscape design, architectural design, and other fields, and has strong quantitative rationality and scientificity. In this study, the SD method to analyze the experience of troubled children in the SOS children's village. The average value of children's perception of outdoor activities was calculated as the average value of the score of outdoor activities, which is the basis of the arithmetic evaluation of children's perception of outdoor activities. $\bar{\text{x}}=\frac{x_{1}+x_{2}+x_{3}+...+x_{n}+}{n}=\frac{\sum_{i=1}^{n}xi}{n}$. After the average value was obtained, we performed the evaluation analysis. The positive value of the score indicates that the score is better, and the larger the value, the more positive it is, suggesting that the children in distress have a higher overall evaluation of the spatial factors of outdoor public activities in the welfare community. A negative value signifies a poor score; the smaller the value, the more negative it is, indicating that children in distress have a poor overall evaluation of the spatial factors of outdoor public activities in welfare communities. We used SPSS software to conduct *t*-test on the obtained data and quantitative analysis to determine the psychological perception of children in distress in the public activity space of the welfare community. During the experiment, the test value of the single-sample *t*-test was set as the middle value of the semantic measurement scale 0 (i.e., subjects had no obvious psychological perception tendency toward the evaluated public space). When the *P*-value in the analysis result is <0.05 (i.e., above the 95% confidence estimation level), it is proved to be significant. The mathematical formula of single-sample *t*-test quantity: $t=\frac{\bar{x}-\mu }{\frac{{}^{\sigma }x}{\sqrt{n}}}$ (where = 1, 2, 3…, *N*; is the sample average, is the population average, is the standard variance, and *N* is the number of samples. Based on the selection of evaluation factors for the physiological and psychological needs of children in distress, summarizing the relevant research literature on outdoor activity space for children in distress revealed that the researchers primarily focused on the physiological and psychological needs of children in distress, including six aspects: ① hearing, ② vision, ③ tactile, ④ interaction, ⑤ safety, and ⑥ education. Thus, this study starts from these six needs. In addition, the environmental support provided by the SOS children's village for children in distress was selected as the main evaluation factor.

## Results

Among 83 children in distress, 43 were boys and 40 were girls. The minimum value of the total score was 27, while the maximum value was 69. The self-awareness test results of 83 children in the SOS children's village were compared with the Hunan norm (see Table 2 for details).

**Table 2:** Comparison between 83 children in the children's village and Hunan norm (*x* ± *s*).

**Factor**		**I Behavior**	**II Intelligence and school**	**III Physical appearance**	**IV Anxious**	**V Gregarious**	**VI Happiness and satisfaction**	**Total score**
Children in distress	SOS children's Village (*n* = 83)	11.57 ± 2.76	8.29 ± 3.44	6.62 ± 2.47	8.20 ± 2.92	8.16 ± 2.26	6.63 ± 1.92	49.51 ± 15.77
Ordinary children	Hunan norm (*n* = 864)	11.97 ± 2.96	8.89 ± 3.27	6.40 ± 2.81	9.35 ± 2.85	8.49 ± 2.20	7.12 ± 1.89	51.98 ± 15.98
*T*		−1.141	1.014	0.763	−3.127	−0.798	−1.729	−1.658
*P*		0.249	0.314	0.442	0.384	0.384	0.086	0.100

*x ± s represents the mean ± standard deviation*.

### Result Analysis 1

Compared with the Chinese urban norm, the scores of boys' behavior factor and self-consciousness were lower than those of intelligence and school situation, while the scores of satisfaction factor were lower than those of the Chinese urban norm. The intelligence, behavior, happiness and satisfaction factor scores, anxiety factor scores, and total scores of girls were lower than those of the Chinese urban norm (Table 3).

**Table 3:** Comparison between the SOS children's village's children in distress and Chinese urban children (*x* ± *s*).

**Factor**	**Gender**	**SOS children's village (*n* = 83)**	**Chinese urban norm (*n* = 846)**	***T***	***P***
I Behavior	Male	11.28 ± 2.62	11.94 ± 2.66	−1.983	0.055
	Female	11.96 ± 2.73	12.88 ± 2.35	−1.297	0.119
II Intelligence and school	Male	9.69 ± 3.66	10.73 ± 3.12	−2.119	0.041
	Female	10.28 ± 3.22	11.05 ± 3.24	−3.722	0.001
III Physical appearance	Male	6.89 ± 2.60	7.98 ± 2.89	−3.096	0.004
	Female	6.34 ± 2.35	7.97 ± 2.08	−3.697	0.001
IV Anxious	Male	8.36 ± 2.94	9.41 ± 2.54	−1.779	0.080
	Female	8.13 ± 2.91	9.20 ± 2.54	−2.451	0.019
V Gregarious	Male	8.20 ± 2.42	8.44 ± 2.01	−1.137	0.263
	Female	8.64 ± 2.08	9.13 ± 1.78	−1.727	0.093
VI Happiness and satisfaction	Male	6.69 ± 1.98	7.56 ± 1.65	−2.418	0.020
	Female	6.68 ± 1.89	7.64 ± 1.65	−3.109	0.000
Total score	Male	51.11 ± 16.22	56.62 ± 9.91	−3.998	0.000
	Female	52.03 ± 15.18	57.26 ± 9.66	−4.910	0.000

*x ± s represents the mean ± SD, and t is the value of the t-test, which is used to compare the difference of the mean between the two groups of data; P is the indicator of decreasing reliability of results*.

### Result Analysis 2

The comparison of EPQ scores of children of different genders and their comparison with the corresponding national norm revealed that the scores on the extroversion scale of boys and girls were lower than the national norm, and the difference was statistically significant (*P* < 0.05). In addition, the score on the mental quality scale for girls was lower than the national norm. The comparison demonstrated that the score of the mental quality factor for boys was higher than that for girls, and the score of the concealment scale for boys was lower than that for girls (*P* < 0.05; Table 4).

**Table 4:** EPQ scores of children in the children's village and comparison with national norm (*x* ± *s*).

	**P (spirituality)**	**E (inward and outward)**	**N (emotion)**	**L (concealment)**
**Different gender**				
Boys group	4.69 ± 3.18	16.95 ± 3.99	9.23 ± 4.97	12.90 ± 4.18
Girls group	1.92 ± 1.52	16.11 ± 4.70	10.16 ± 5.41	14.54 ± 4.13
*P*	0.000	0.283	0.301	0.021
**Male**				
SOS children's village children's group	4.69 ± 3.18	16.95 ± 3.99	9.23 ± 4.97	12.90 ± 4.18
Norm group	5.32 ± 2.98	18.32 ± 3.75	8.53 ± 4.68	13.89 ± 4.38
*P*	0.225	0.038	0.384	0.146
**Female**				
SOS children's village children's group	1.92 ± 1.52	16.11 ± 4.70	10.16 ± 5.41	14.54 ± 4.13
Norm group	3.78 ± 2.40	18.15 ± 3.56	8.60 ± 4.90	15.81 ± 4.30
*P*	0.000	0.012	0.087	0.070

### Result Analysis 3

The analysis of each factor score, the total score of self-consciousness, and Eysenck scale demonstrated that the total score of PHCSS significantly negatively correlated with the scores of EPQ psychoticism and neuroticism, as well as factor scores of behavior, intelligence, and school situation, mental quality, and neurological quality. In addition, the gregarious factor score and happiness satisfaction factor negatively correlated with the mental quality and neurological quality scale, but significantly positively correlated with introversion and introversion and concealment scale. A significant positive correlation was observed with the masked subscale. Intelligence and school situation positively correlated with the introversion and introversion subscale because of physical appearance and attribute factor scores, while anxiety factor negatively correlated and positively correlated with neuroticism and concealment factor scores, respectively, and positively correlated with introversion and introversion and concealment subscale (Table 5).

**Table 5:** Results of correlation analysis between self-awareness and EPQ scales and children's general situation (*n* = 83).

	**P (spirituality)**	**E (inward and outward)**	**N (emotion)**	**L (concealment)**
I Behavior	−0.475	0.032	−0.366	0.403
II Intelligence and school	−0.263	0.362	−0.403	0.465
III Physical appearance	−0.148	0.602	−0.188	0.126
IV Anxious	−0.188	0.186	−0.505	0.237
V Gregarious	−0.357	0.235	−0.367	0.292
VI Happiness and satisfaction	−0.345	0.453	−0.450	0.241
Total score	−0.436	0.342	−0.496	0.396

### Selection of SD Evaluation Factors Based on Physiology and Psychology of Children in Distress

#### Visual Environment Requirements

Children are curious about things around them and like bright colors and novel and interesting patterns. Owing to their poor living conditions, most children were in poor mountainous areas or remote and backward villages and had an exploratory attitude toward things around them. Unlike adults, children are more sensitive to the outside world (23). Thus, the outdoor public space of the SOS children's village should consider exploring the needs of children in distress.

#### Auditory Environment Requirements

Most children in distress come from minority inhabited areas in Sichuan, where singing and dancing are predominant cultural customs and recreational activities. Music is the key to awaken their local memory. Malon P. Wells highlighted that nature is very attractive to children. Children solve problems in nature games. Through selection, exploration, and discovery, children can enhance their cognitive and physical development (24). Thus, children are close to nature and have a desire to be close to the sound of insects, birds, and water.

#### Tactile Environment Requirements

Tactile environment requirements denote the physical environment that children can perceive physiologically. Owing to the lack of awareness of the things around them, they are used to measuring and perceiving themselves. When they play in the outdoor space, they are attracted by the vegetation odor form. Exposure to natural elements and experience of the natural environment can not only stabilize emotions, augment attention, promote the development of children's cognition, and balance coordination ability but also promote social interaction, increase the frequency of children's contact with the outside world, and enhance creativity (25). By touching natural elements closely in the outdoor space, children in distress can make this soft vegetation or object liberate their body and mind, smooth and relieve their emotions, and release their pressure. Real feelings in nature and participation in activities can effectively decrease the disaster risk in growth (26).

#### Interactive Environmental Requirements

Interactive environmental requirements are embodied in activity dependence, including active participation, experience, and education. The pathological psychological problems of children in distress are resolved to some extent in the interactive space, which can effectively help children in distress alleviate problems such as autism and loneliness (27). Ordinary children display dependence on their parents in outdoor activities alone. Children in distress are not easy to exhibit trust in their parents; instead, they show a state of being clever, calm, and lonely in outdoor activities alone. In group activities, they exhibit dependence on their peers, lively, chasing, and playing. owing to curiosity and exploration, the activity places for children in distress are more concentrated in “informal” places like greening on both sides of the road and large green open spaces (28).

#### Safety and Environmental Requirements

The vital factor in the activities of children in distress is safety (29), as they have experienced adverse events and psychological and physiological injuries before entering the village. Thus, safety and comfort should be considered in the outdoor activity space. Some safety protection facilities and buffering soft small sandpits should be provided for facility accessories.

#### Children's Intellectual Needs

Children in distress in the park are usually aged 2–10 years. At this stage, they are full of curiosity and exploration of the outside world. Their strong thirst for knowledge enables them to constantly learn knowledge and accrue survival and life experience in activities. Hence, educational activities should be considered in the activity sites in the park.

#### Comprehensive Analysis

Combined with the physiological and psychological characteristics of children in distress, this study determined nine pairs of adjectives to reflect the needs of children in distress: color richness (plain—rich); light in the village (more—less); water flow sound (more—less); insects singing and birds singing (noisy—quiet); plant season (rich—single); vegetation growth (lush—sparse); touchability (very strong—very weak); the sketch device is entertaining (interesting—boring). In the study of safety problems during play (hidden danger—safety), the subjective assessment is categorized into five levels, which are distinguished by “very,” “general,” and “none.” The abovementioned adjectives are divided according to the evaluation level from left to right, and assigned −2, −1, 0, 1, and 2, respectively. In this study, 83 troubled children in the SOS children's village were selected for the test. Before the test, the evaluation factors were introduced to the experimenter, the requirements were explained, and the relevant factors in the form were evaluated per the standards. A total of 83 questionnaires were collected, of which 80 were valid.

### Summary of the Spatial Plane Model of the Children's Village

**Figure 4:**
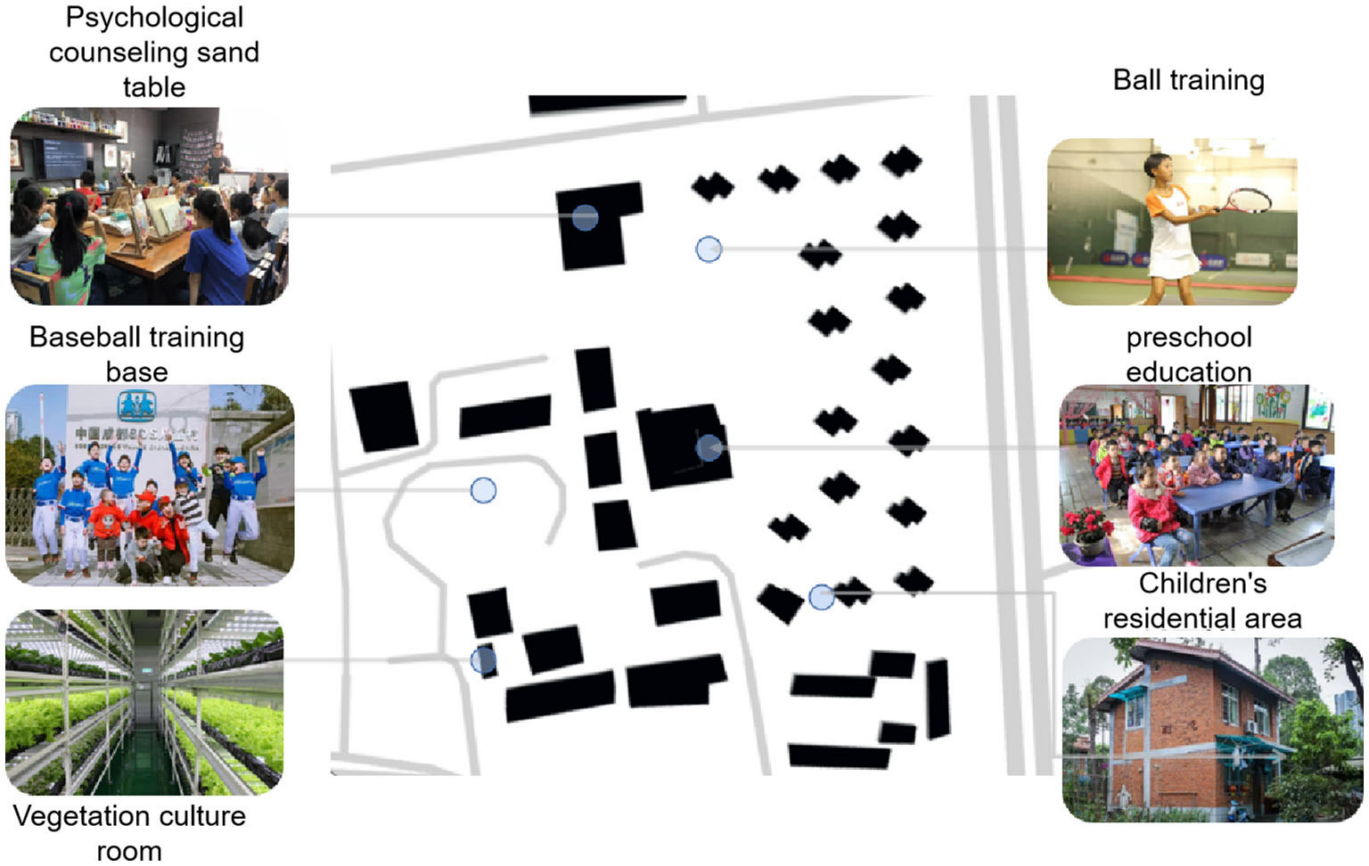
Summary of the plane model of children's village.

According to Piaget's classification, children's game behavior was categorized into functional games, constructive games, hypothetical games, and regular games (30). Research on appropriate outdoor play activities is conducive to muscle strength, psychological patience, and cardiovascular health of children in distress (31). Thus, the plane organization mode of the SOS children's village was summarized, which was categorized into four main spaces: a large toy fun pool, cement playground, fitness equipment area, and plastic playground. Furthermore, four plane organization modes were found to affect the activities of children living in the SOS children's village (Table 6, Figure 4).

**Table 6:** Space classification of several important outdoor activities in the SOS children's village.

**Site type**	**Age**	**Characteristic**	**Scene status**
A: Large toys, children's life fun pool	3–6 years	Younger children cannot consciously regulate and control their activities but can play interesting games under their parents' care. Thus, intellectual + physical activity facilities with both fun and exercise are selected, and equipped with a rest corridor for caregivers	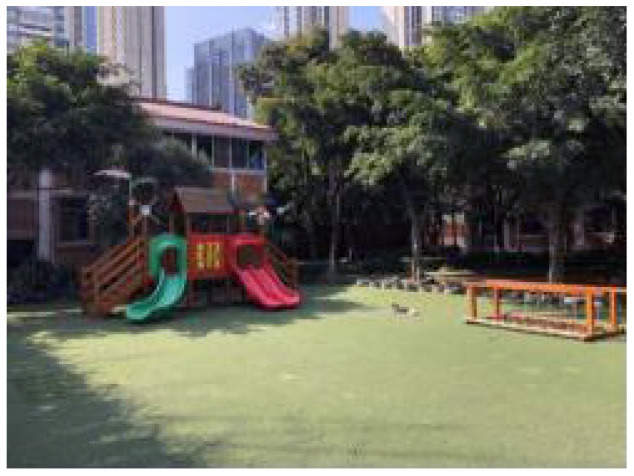
B: Cement playground (basketball court, tennis court, badminton court)	8–12 years	The area is flat and spacious, equipped with basketball racks and nets to effectively exercise children's physical strength and teamwork ability	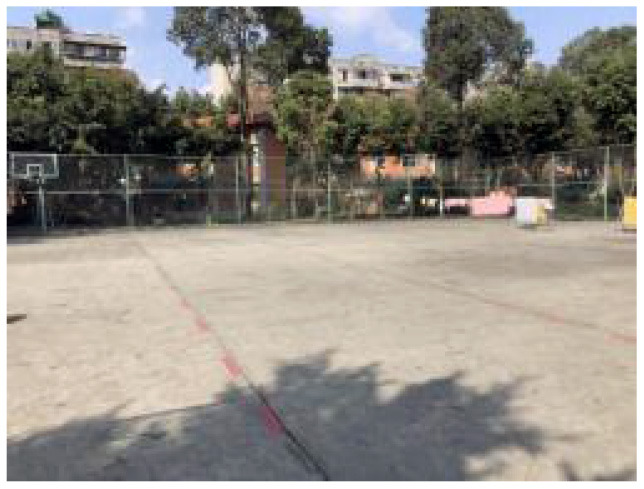
C: General hard fitness equipment area	6–12 years	The area belongs to children's physical fitness coordination and other exercise areas, equipped with various activity equipment, including rope, chain, slide, and swing	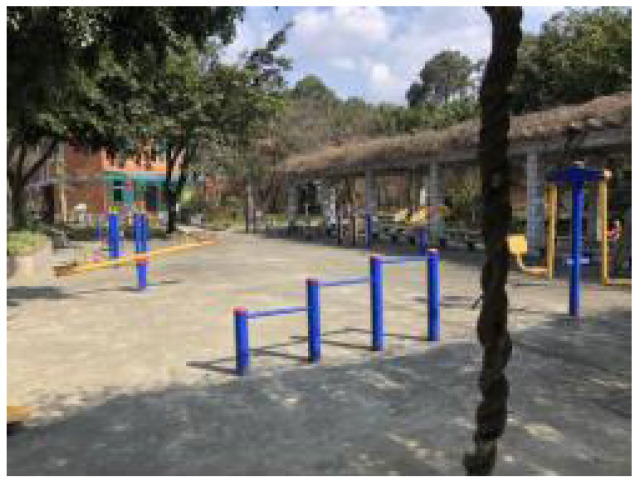
D: Lawn playground (football field, baseball field, plastic track)	8-12 years	A circle around the playground dominated by football field is equipped with 200-m red runway and rest seats. The stadium runway is used for children's training and seats for rest	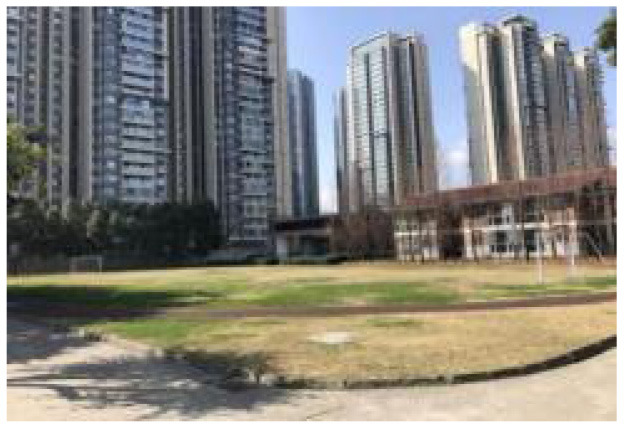

#### A Space Mode—Children's Fun Pool

The children's fun pool is a plastic activity site with an activity area of 100 m^2^. The main users' children aged 3–6 years, who are young and have poor sports ability. They like to play in this auxiliary amusement facility surrounded by venues. Children's fun pool can effectively attract children of this age and let them break barriers and participate in activities. The site is equipped with children's outdoor large toys. The interior is paved with a plastic lawn, which is hard as a whole. In hot summer, it emits the smell of plastic because of exposure to the sun. It is mostly covered with ginkgo trees, Magnolia, climbing rattan, and other vegetation. The configuration type of vegetation is relatively single, but the arrangement of climbing rattan is continuous, which can create a continuous section. The southeast portion is close to an 80-m Boulevard full of vines. In summer, it is a path where children like to gather and enjoy the cool.

#### B Space Mode—Cement Playground

The cement playground is a relatively wide playground with an area of 400 m^2^ in the children's village, including a basketball court and tennis court, surrounded by a circle of green barbed wire. Children who come to this activity are >8 years old and have certain motor ability and limb coordination ability. Children in this age group are in a crucial period of physical growth, and their limb coordination abilities and activity ability are basically formed. As the site is made of cement, it is hard. Owing to the wind and rain in the open air all year round, the court lines on the site are worn, and the ball baskets and nets are loose and damaged. In winter and summer, the activity is reduced owing to the outdoor temperature.

#### C Space Mode—Fitness Equipment Area

Simple fitness equipment stands on the hard cement ground, including a swing and seesaw, which is no different from the equipment for outdoor public activities in general residential areas. Typically, children come here for less time. The equipment color is a combination of blue and yellow, and there is no sign of wear and aging. On the south side of the field is a tree-lined corridor full of climbing vines, which is connected with the cement playground in the north. Besides, there is less vegetation around.

#### Class D Plane Function Space Model—Lawn Activity Field

A small football field with an area of 350 m^2^ is surrounded by red plastic runways. These areas are primarily established for baseball or football games. Children exercise body and cooperation ability in communication and confrontation with their peers. The lawn in this area is planted with general natural turf. The seasonal phase of turf changes because of seasonal changes. The applicable crowd of the stadium is usually children aged >8 years.

## Discussion

### Result Analysis of Psychological Problems

Through analysis, the self-awareness level of children in the SOS children's village was found to be lower than that of children in China. These children usually lack a sense of happiness and security, have low self-satisfaction, have relatively negative emotions, and are prone to negative emotions like anxiety and depression. The formation of self-consciousness correlates with the environment in which they were born and grew up, and the reasons for the formation of their bad emotions are as follows: ① troubled children in the SOS children's village have been concerned by the surrounding people for a long time, and their psychology has developed a special group label. The impact of this brand enlarges their various behaviors in life and hinders the correct formation of children's self-consciousness. ② Most children in distress come from poor mountainous areas in Southwest China. Before entering the village, they have experienced vicious negative events such as family fragmentation, parents' death, and relatives' abandonment. These events lead to physical and psychological trauma and hinder the development of their self-consciousness. ③ Children in the SOS children's village are introverted and withdrawn, which influences their ability to communicate with others or impart knowledge. Thus, most children in the children's village do not like learning, and their poor grades also lead to the imperfect development of their self-consciousness. Children in the children's village tend to be introverted, lonely, and not good at talking to others. Moreover, children in trouble like quiet daily life. Male children are more withdrawn and rebellious, with poor environmental adaptability, poor emotion, and poor self-regulation ability, while girls are timid, strongly dependent on others, and have dull and stereotyped behavior. With the extension of time in the village, children's concealment decreased and their simplicity improved. Furthermore, the study demonstrated that individual children had a concealment score of >79%, had a tendency to disguise, liked to disguise themselves, and had poor simplicity.

### Result Analysis of Physiological Problems

**Figure 5:**
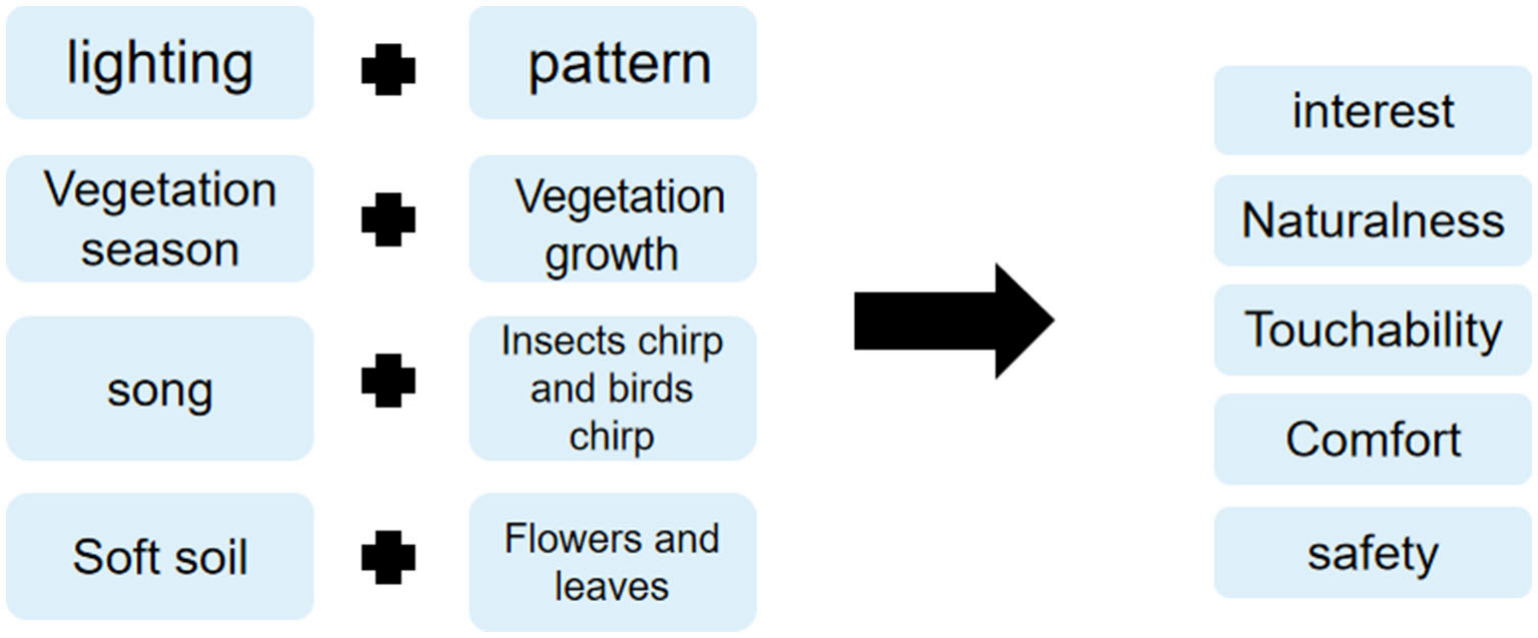
Characteristic diagram of SD factor.

By summarizing and classifying the scores of 80 questionnaires, the SD curve was obtained, and the longitudinal intergroup comparison, that is, the score comparison of different plane modes, was conducted to obtain the advantages and disadvantages of the plane form (Figure 5).

### Evaluation and Analysis of Spatial SD Factor Combination

**Figure 6:**
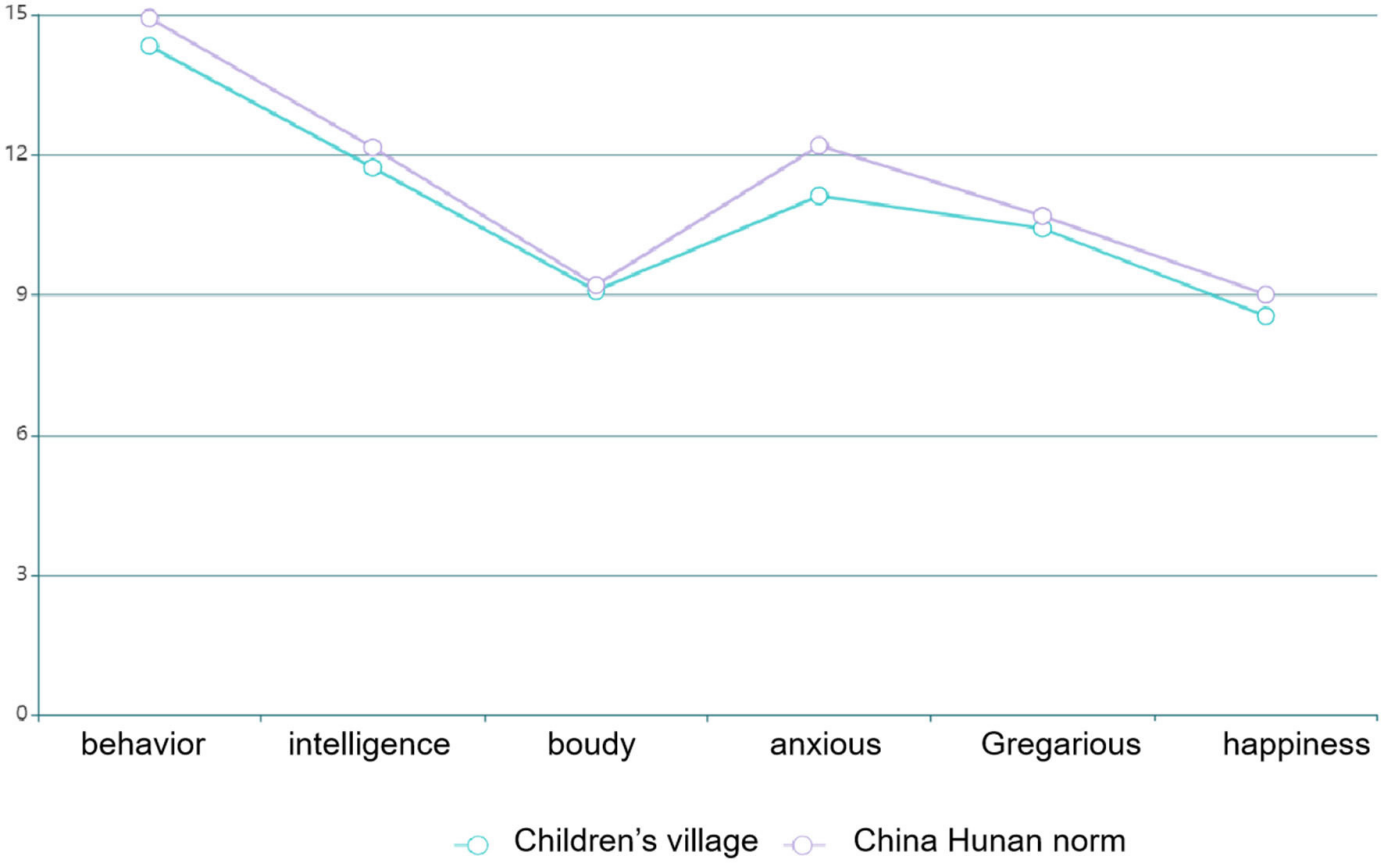
Comparison between PHCSS scores of children in distress in Chengdu SOS children's village and Chinese norm scores.

**Figure 7:**
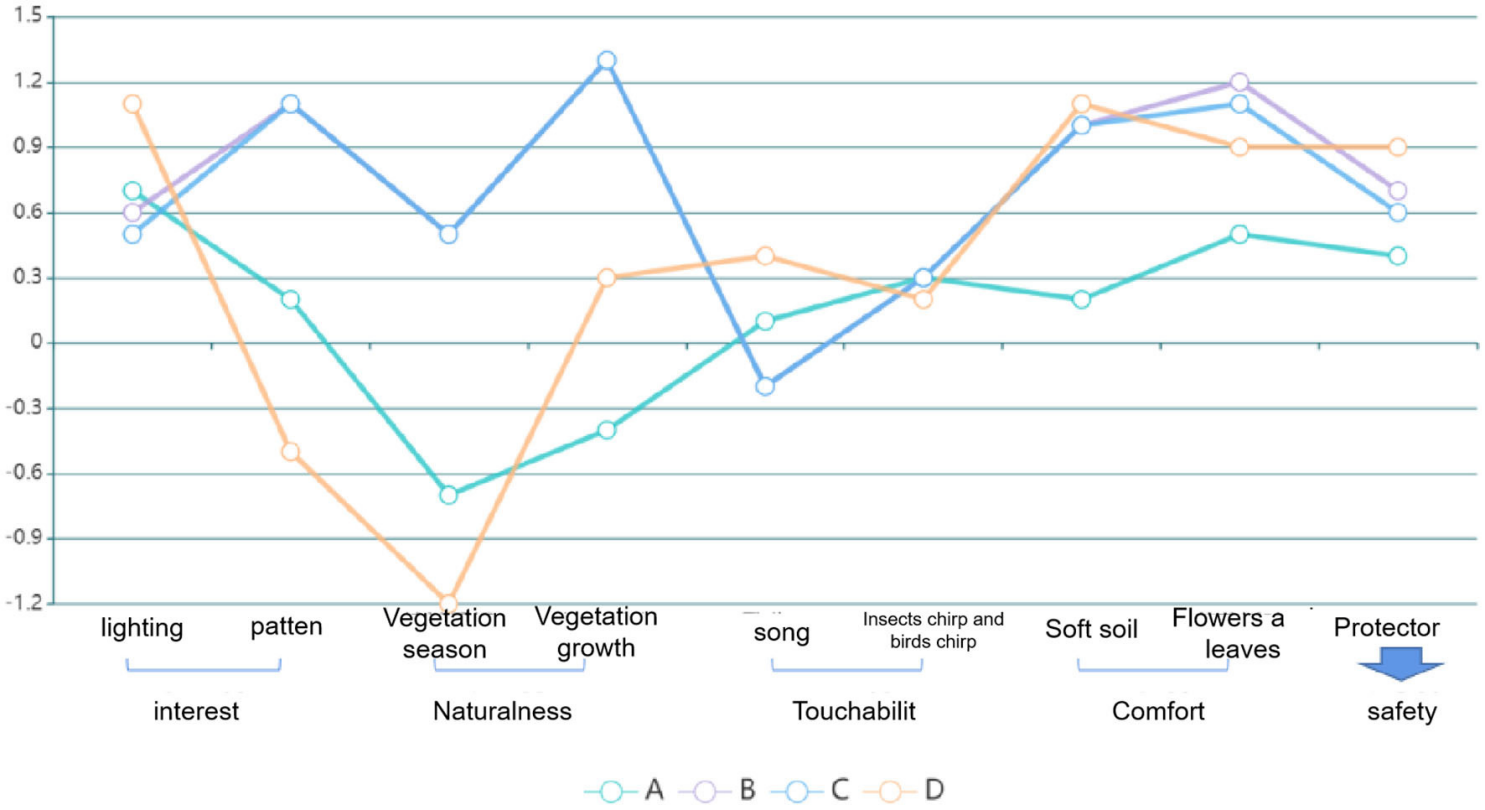
Comparative analysis of PHCSS-SD scores between activity space pattern groups.

According to the self-awareness score and spatial factor extraction of children in distress, the PHCSS-SD quantitative analysis method was formed, and the design strategies in the process of outdoor space communication activities to solve the psychological problems of children in distress were analyzed (Table 7, Figures 6, 7).

**Table 7:** SD factor example classification table.

**Criterion layer**	**Detailed rules and regulations**	**Example**
Interest	Lighting	Spot, light, spot
	Interesting pattern	Wall painting, blackboard newspaper, stone wall painting
Naturalness	Vegetation season	Wall painting, blackboard newspaper, stone wall painting
	Vegetation growth	Luxuriant and sparse appearance
Comfort	Song	Radio, music, reading sound
	Insects singing and birds singing	Birds, sounds
Touchability	Soft soil	Soil quality and hardness
	Flowers and trees	Category, touch, smell
Security	Protector	Isolation zone, buffer zone, sand pit

#### An Activity Space Model

From the broken line of type A activity space, it can be deduced that the anxiety factor score of target children is high in creating the visual environment of “interesting pattern” and “daylighting.” Thus, it is appropriate to increase the thought-provoking experience in the activity—the large toy space and children's interesting pool of type A plane space model have advantages so that it can better create this environment. As this region mainly aims at children in difficult circumstances, the safety requirements are high. In the plant season construction, it is a large deciduous vine, which makes the planting of type A plane space model vegetation more advantageous than that of single tree row planting. Besides, the advantages of planting shrubs are higher than that of trees in terms of touchability, which can create a closer natural experience for children in distress.

#### B Activity Space Model

Type B space has a large open space, which is similar to area A in terms of “daylighting” requirements, to facilitate lighting and calcium supplements for children's activities. Regarding the requirements for “beautiful songs,” as most of the activities are large-scale sports activities and need to be stimulated by relevant music, the requirements for hearing are high. Meanwhile, because children in distress are still young and lack understanding of the world, safety is paramount. The surrounding vegetation can better decrease the interference of vehicle space to the open space, create a safer and free environment, and optimize the activity experience of children in distress.

#### C Activity Space Model

Except for “corridor lighting” and “beautiful song,” all scores of the SD curve in mode C were high. As this corridor comprises vines, it reflects the core through rich planting methods and large green space width and has stable ecology and good ornamental. Thus, the equipment activity space besides the corridor also forms a relatively natural and comfortable playing space and ornamental walking space (32, 33) so that children in need can make space choices based on their needs.

#### D Activity Space Model

Type D space has the largest soft artificial grassland for children's daily activities, which is in contrast to the hard ground in area B. As it is an artificial turf football field, the single score of “vegetation season” is the lowest, and the “vegetation growth” is relatively weak, but the “education” brought by large-scale activities is high.

### Strategy

Based on the field investigation and SD psychological measurement analysis of the public space and troubled children in the SOS children's village in Chengdu, this study establishes the potential relationship between the outdoor public space and the physiological and psychological growth and development of troubled children, as well as provides some suggestions for the construction of community outdoor public space.

1) In the plane organization of outdoor public space, the division should not be too noticeable.
2) Enrich the terrain changes and enhance the interest in the activity space.
3) When there are two or more planting spatial patterns, the primary–secondary relationship should be created and should not be homogeneous.
4) When constructing the activity place, consider the safety and education brought by the activity place.
5) Try to create a transition space between spatial modes.
6) Properly control the sound scene of the outdoor space.
7) Add interesting patterns.

## Conclusions

This study provides a theoretical basis for developing children's welfare in China. With the acceleration of urbanization in China, community public outdoor activity space has become one of the most broadly used public spaces for children. Nevertheless, at present, the activity functions and activity facilities for children in the public space are single lack attraction and richness. As mentioned in the first China Children's Friendship Action Seminar, children's friendships should be reflected in the details of life scenes and should be transformed from grand narrative planning to the corner of life. Thus, how to stimulate the strong sense of belonging and self-identity of children in distress and effectively address the psychological problems should be explored. Activity space optimization creates a comfortable and safe activity space for children in distress, which is of great and practical significance to help them thrive.

## Data Availability Statement

The original contributions presented in the study are included in the article/supplementary material, further inquiries can be directed to the corresponding author/s.

## Ethics Statement

The study was approved by Southwest Jiaotong University (approval date: January 20, 2022) and involved human research. Ethics approval and written informed consent were not required for this study in accordance with national guidelines and local legislation.

## Author Contributions

FF and XL: resources. FF: supervision and writing—review and editing. XL: validation and writing—original draft. All authors contributed to the article and approved the submitted version.

## Funding

This research was supported by the annual project of the National Social Science Foundation of China (19BSH101), the Ministry of Humanities and Social Sciences in Western China (18XJCZH003), the Center on Child Protection and Development, Sichuan (ETBH2021-ZD001), and the Technological Innovation R&D Project from the Chengdu Science and Technology Bureau in 2022 (2022-YF05-00297-SN).

## Conflict of Interest

The authors declare that the research was conducted in the absence of any commercial or financial relationships that could be construed as a potential conflict of interest.

## Publisher's Note

All claims expressed in this article are solely those of the authors and do not necessarily represent those of their affiliated organizations, or those of the publisher, the editors and the reviewers. Any product that may be evaluated in this article, or claim that may be made by its manufacturer, is not guaranteed or endorsed by the publisher.
